# The key roles of teammates, coaches, and instrumental support in adolescent sports participation: a one-year prospective study

**DOI:** 10.3389/fspor.2025.1499693

**Published:** 2025-01-20

**Authors:** Gwennyth E. Spruijtenburg, Femke van Abswoude, Sebastiaan W. J. Platvoet, Mark de Niet, Bert Steenbergen

**Affiliations:** ^1^ Behavioural Science Institute (BSI), Radboud University, Nijmegen, Netherlands; ^2^ Institute for Studies in Sports and Exercise, HAN University of Applied Sciences, Nijmegen, Netherlands

**Keywords:** organized sports, youth, social support, sources of support, types of support, longitudinal design

## Abstract

**Introduction:**

Sports participation is important for promoting lifelong health and well-being. However, it often declines during adolescence, highlighting the need to understand the factors related to adolescent sports participation. This prospective study examines the associations between different sources (e.g., family, peers, coaches, teachers) and types (e.g., emotional, instrumental, co-participation) of social support and hours of organized sports participation among secondary school students (*N* = 294). It also explores whether these associations change from the second to the third year of secondary education.

**Methods:**

Data were collected using questionnaires and anthropometric measurements in October/November 2021 and October/November 2022. Multilevel linear fixed effects regression models were used to analyze relationships between sources and types of social support and sports participation. Additionally, scatterplots illustrated individual variability in these associations.

**Results:**

Results showed significant associations for various sources and types of social support with organized sports participation. Social support from teammates and coaches and instrumental support emerged as the strongest predictors of hours of participation. Additionally, we found that the relationships remained stable over time. Yet, substantial individual variability in how social support related to sports participation was also observed.

**Discussion:**

These findings emphasize the importance to promote social support from within the sports environment and to encourage instrumental support. Tailored approaches addressing individual differences are recommended to enhance adolescent sports participation.

## 1 Introduction

An active lifestyle, particularly through sports, is essential for lifelong health and well-being (1–4). Despite its benefits, sports participation often declines from adolescence onwards (5–8), which highlights the need to understand and address the factors that influence sports participation of adolescents. Consistent with recent reviews (9–12), our previous research has confirmed the importance of factors such as motives and perceived competence in organized sports participation during adolescence (13, 14). Our findings also supported the notion that social support may play a crucial role alongside these factors. However, the role of social support remains a largely unexplored area of research. This is surprising given the developmental changes during adolescence, where relationships with parents and peers evolve (15, 16). In the present study, we will examine the role of social support in organized sports participation using a prospective design.

Social support for sports participation can be provided by different sources within one's social network (e.g., parents, siblings, peers, teachers, coaches) and through different types of support (e.g., emotional, esteem, informational, instrumental, co-participation, modelling) (17, 18). Thus far, studies on social support have either examined total social support (which is expressed as a composite score) or only one or a few of the sources or types of social support (18–26). It was evident from these studies that various sources and types of social support can influence physical activity levels (17, 27–29). However, the relative importance of these sources and types of social support varies, with some exerting more influence than others (17, 27). Regarding sources of social support, positive relationships with sports participation are most commonly reported for support from parents, peers and coaches (19–21, 24, 30), while physical education teachers and siblings have a less pronounced influence (26, 27, 31). Regarding types of social support, emotional and instrumental support appear to be the most important (17, 27). Despite these valuable insights, no studies have yet examined the combined role of a wide range of sources and types of social support in adolescent sports participation. By addressing this gap, this study aims to better understand how different sources and types of support simultaneously relate to levels of sports participation.

In addition, it is essential to explore how the relationships between social support and sports participation evolve over time. The relative importance and influence of different sources and types of social support can shift as adolescents grow older (32, 33). For example, while parental support may be particularly crucial for physical activity during early adolescence, peer influence tends to become more significant as adolescents mature (32). As another example, the types of parental support that affect sports participation are likely to evolve across developmental stages (33). Specifically, the role of parents gradually often shifts from actively encouraging their children to explore various sports during childhood to becoming more passive supporters who facilitate optimal training conditions during adolescence. These findings highlight the dynamic nature of social support during adolescence and emphasize the need to consider these changes over time. Given that most studies to date have used a cross-sectional methodology (17, 19, 23, 24, 27, 30), there is a clear need for more prospective research (even over short periods). This study seeks to contribute to understanding whether and how social support and sports participation of adolescents change over time, providing insights that could inform the development of age-appropriate programs tailored to their changing needs.

In summary, this study will add to our knowledge of adolescent sports participation by (1) addressing the underexplored role of social support in organized sports participation among adolescents, (2) examining a wide range of sources and types of social support simultaneously, rather than focusing on composite scores or a limited amount of sources or types, and (3) employing a prospective design to capture how the relationships between social support and organized sports participation evolve over time. These insights can inform the development of more targeted strategies to sustain sports participation and foster lifelong health and well-being. Specifically, we examine the association between different *sources* (i.e., father, mother, siblings, friends, teammates, coach, and PE teacher) and *types* (i.e., emotional, esteem, informational, instrumental, co-participation, modelling) of social support and hours of organized sports participation among adolescents in both their second and third years of secondary education (*N* = 294). Whereas other studies primarily focus on outcomes related to sports participation (e.g., intention to continue) or to a binary measure of whether or not adolescents participate in sports (e.g., (19, 24), the present study focuses on the total time spent in organized sports. This approach provides a more nuanced understanding of sports participation and its associated factors. We hypothesize that social support from parents, peers (i.e., friends and teammates) and the coach will be most strongly positively related to hours of sports participation. In addition to this, we hypothesize that various types of social support will be positively related to hours of sports participation, with emotional and instrumental being the most influential types. As a final and more exploratory research question, we examine whether the associations between the different sources and types of social support and hours of sports participation change over time.

## 2 Materials and methods

### 2.1 Study design

This longitudinal study is part of the Transitions Into Active Living (TRIAL) project, which investigates changes in physical activity during key life transitions. We followed secondary school students from the start of their first year to the start of their third year of secondary education. Data were collected in seven waves between October 2020 and November 2022. For the current analyses, we used data from Wave 4 (October/November 2021) and Wave 7 (October/November 2022), corresponding to the start of the second and third years of secondary education. From here on, we will refer to Wave 4 as “Year 2” and Wave 7 as “Year 3”. This study was approved by the Ethics Committee of the Faculty of Social Sciences at Radboud University (ECSW-2020-107).

### 2.2 Study sample

We selected a convenience sample of eight secondary schools with which our research team had established working relationships. These schools were located in two provinces in the eastern Netherlands. In June 2020, we informed the physical education teachers via email and invited them to participate with their schools. Five schools agreed to participate. Subsequently, we sent information letters and informed consent forms both on paper and via email to all first-year students (*n* = 1,127) and their parents. The recruitment period for this study spanned from September 9, 2020, to the end of October 2020. Of the potential participants, 530 adolescents (50.9% boys) received parental consent to participate and were also asked to provide their own written consent. We included adolescents in the analysis sample if they had complete data on sports participation and all social support variables at both Year 2 and Year 3.

### 2.3 Assessments

We used a questionnaire and anthropometric measurements to assess the variables relevant to the present study. The questionnaire measured organized sports participation, social support, motives, perceived competence, age and ethnicity. Although additional variables were included, they were not used in the current analyses. The full version is available at: https://doi.org/10.17026/SS/LA3KIZ. Anthropometric measurements (height and weight) were taken to calculate BMI. The variables relevant to the present study are described in detail below.

#### 2.3.1 Background measures

At the start of the study (Year 1), we collected data on adolescents' age, gender, and ethnicity through a questionnaire. Students self-reported their age and gender. Ethnicity was determined based on the definition of Statistics Netherlands (34, 35). Adolescents reported their parents' countries of birth, and we categorized them native Dutch if both parents were born in the Netherlands (34), or non-native Dutch if at least one parent was born outside the Netherlands (35). In addition, we determined BMI through anthropometric measurements conducted at Year 2 and Year 3. Trained research assistants measured height using a Seca stadiometer and weight with a Seca scale. BMI (kg/m²) was then calculated from these measurements.

#### 2.3.2 Organized sports participation

We defined organized sports as any sport practiced in clubs (e.g., soccer and tennis) or fitness centers, or led by a trainer or coach (e.g., boot camp). Organized sports participation was assessed using items that were based on the validated Flemish Physical Activity Questionnaire (FPAQ) (36). Adolescents were first asked if they participated in sports. If yes, they listed up to three sports, and for each sport, they indicated if they practiced it in a club/fitness center or under supervision. A sport was classified as organized if either condition was met. Weekly hours of participation were measured for up to three organized sports and summed to calculate total weekly hours of organized sports participation. Non-participants were assigned zero hours. Two outliers (i.e., 90 and 230 h) were excluded as they were deemed implausible (the highest remaining value was 24 h).

#### 2.3.3 Social support

We assessed seven sources (i.e., father, mother, siblings, friends, teammates, coach, and PE teacher) and six types (i.e., emotional, esteem, informational, instrumental, co-participation, modelling) of social support using 14 items. For each item, students were presented with a list of the seven potential sources of social support and instructed to select all sources that applied to them. Three items each represented emotional, esteem, informational, and instrumental support, while one item represented co-participation and another item represented modelling (see Table 1). The items regarding emotional, esteem, informational, and instrumental support were adapted from validated questionnaires on social support and sport (28, 29). The items regarding co-participation and modelling were developed based on a key systematic review (17). For each source of support, we calculated the total number of items that were selected (overall score 0–14). For each type of support, we calculated the average number of sources from whom social support was perceived (overall score 0–7).

**Table 1:** Types of social support and items in questionnaire.

Type of social support	Item
Emotional support	1. Who encourages you to exercise/play sports?
	2. Who talks to you about exercising/playing sports?
	3. Who makes you feel like they are always there for you?
Esteem support	4. Who gives you the confidence to exercise/play sports?
	5. Who tells you what your athletic abilities are?
	6. Who tells you that you are good at exercising/playing sports?
Informational support	7. Who gives you advice about exercising/playing sports?
	8. Who helps you decide what to do?
	9. Who gives you tips to improve in exercising/playing sports?
Instrumental support	10. With whom do you go to the location where you can play sports?
	11. Who helps you plan when you can exercise/play sports?
	12. Who pays for the equipment and/or memberships so that you can play sports?
Co-participation	13. Who plays sports with you?
Modelling	14. Who is your role model for exercising/playing sports?

#### 2.3.4 Covariates

Previous research indicated that sports participation is influenced by a number of factors in addition to social support (9–12). In line with the literature, we recently showed that motives and perceived competence were important factors in this study sample (13, 14). Therefore, we included these two factors as covariates in our analysis. Hereby, this paper gives insight in how different sources and types of social support relate to one another and to other relevant factors with regard to sports participation.

Perceived competence was assessed using a modified version of Harter's perceived competence scale (37). We selected and adapted six items of the physical domain. The final statements were: “I do very well at sports”, “I wish I were a lot better at sports”, “I do well in any new sports that I have not tried before”, “I feel that I am better than peers my age at sports”, “In sports, I usually watch instead of participate”, and “I do not do well in new sports”. Participants responded to these statements on a 4-point Likert scale ranging from 1 (totally disagree) to 4 (totally agree). The average score of these responses, including the reversed scoring for the second, fifth, and last statements, provided the overall perceived competence score.

Motives for participating in organized sports were assessed using eight items on a 4-point Likert scale (1 = totally disagree; 4 = totally agree). Students responded to the prompt, “Why do/would you participate in organized sports?”. These items were based on the Motives for Physical Activity Measure-Revised (MPAM-R) scale and prior studies on adolescent physical activity behaviors (38–41). We included items for five motives: enjoyment, social, competence/challenge, appearance, and fitness. Specifically, the items were: enjoyment (“it is a fun thing to do”), appearance (“to improve body shape”), fitness (“to be healthy”), social (“to meet up with peers”, “friends participate too”), and competence/challenge (“to perform well”, “to be the best”, “to compete with others”). The average of these responses calculated the overall motives score.

### 2.4 Procedures

Data collection procedures are the same as reported elsewhere (13, 14). A team of trained testers collected data during regular physical education classes held in an indoor facility. Students completed the online questionnaire on their laptop computers or smartphone via LimeSurvey. Anthropometric measurements and motor skills tests were also conducted during the same session (motor skills tests were conducted for the broader TRIAL project, but these data are not included in the present study).

### 2.5 Data analysis

All analyses were conducted using Stata software (version 17) (42). Our data had a multilevel structure with repeated measures (level 1) nested within adolescents (level 2) who were clustered in classes (level 3) within schools (level 4) (43). Due to the small number of schools (*n* = 5), however, we could not perform four-level analyses (43). Nevertheless, it remained appropriate to model within-person changes over time using the repeated measures as a separate level, despite the limited number of data waves (43–47). Thus, we accounted for a three-level structure with repeated measures nested within adolescents who were clustered in classes (*n* = 54).

To achieve this, we transformed the data into long format with two measures for each participant, one for Year 2 and one for Year 3. We included a dummy variable, 'school year,' allowing us to model the repeated measures. Additionally, we declared the dataset as panel data nested within classes, aligning with the clustered nature of the data within classes.

To examine the relationships between the sources and types of social support and organized sports participation, we applied multilevel linear fixed effects regression models using the “xtreg” command in Stata. Initially, in Model 1 (the basic model), we included background measures (i.e., age, gender, ethnicity, and BMI) and organized sports participation in Year 1 (yes or no) as predictors of participation in Year 2 and Year 3. Following this, we conducted two series of analyses: one for the sources of social support and another for the types of social support. Each series consisted of five models.

The five models for the sources of social support were as follows: In Model 2a-g, each source of social support was added individually to the basic model. In Model 3, all sources were included simultaneously. In Model 4, covariates (i.e., motives and perceived competence) were added to the previous model. To examine changes in the relationships between the sources of social support and sports participation over time from Year 2 to Year 3. We introduced interaction terms (i.e., father*school year, mother*school year, siblings*school year, friends*school year, team*school year, coach*school year, PE teacher*school year). In Models 5a-g, we added one interaction term at a time. Finally, in Model 6, all interaction terms were included simultaneously. The models for the types of social support followed the same approach.

In addition to the regression models that examined average effects and statistical significance, scatterplots were used to illustrate the variability of these relationships across individuals.

## 3 Results

### 3.1 Sample characteristics

Of the 530 adolescents who had parental consent to participate in the study, 461 adolescents took part in the first measurements of the study (Year 1). By the start of their second year (Year 2), complete data were available for 430 adolescents. By the start of their third year (Year 3), the number decreased to 294, resulting in an analysis sample of 294 adolescents. Common reasons for dropout included repeating a grade, relocating, losing interest in study participation, or being ill on the day of testing.

There were no significant differences in age, gender, ethnicity, BMI and prior organized sports participation between the groups included and excluded from the analysis. However, the analysis sample had a higher proportion of adolescents enrolled in higher general and pre-university educational paths (i.e., higher general, higher general/pre-university, and pre-university) and a lower proportion of those in pre-vocational education paths (i.e., pre-vocational, pre-vocational/higher general, and pre-vocational/higher general/pre-university) compared to the excluded group (*p* < .001). Descriptive characteristics for the background measures of the analysis sample are shown in Table 2. Descriptive statistics for the study variables are shown in Table 3.

**Table 2:** Characteristics of adolescents in the analysis sample (year 2).

	*n*	%	M	SD
Age	282		13.47	0.47
Gender				
Boy	141	47.96		
Girl	153	52.04		
Ethnicity				
Native Dutch	263	89.46		
Non-native Dutch	31	10.54		
BMI	280		18.96	2.66
Organized sports participation in Year 1				
Yes	219	87.95		
No	30	12.05		
Educational paths				
Pre-vocational	31	10.54		
Pre-vocational/higher general	26	8.84		
Pre-vocational/higher general/pre-university	79	26.87		
Higher general	24	8.16		
Higher general/pre-university	71	24.15		
Pre-university	63	21.43		

**Table 3:** Descriptive statistics and comparative analysis of year 2 and year 3 (*n* = 294).

	Scale	Year 2		Year 3		*P*	Effect size
		M	SD	M	SD		
Organized sports participation							
Hours per week	N/A	3.8	2.8	4.1	3.0	.030	0.13
Sources of social support							
Support by father	0–14	7.81	3.77	7.75	3.86	.708	−0.02
Support by mother	0–14	8.04	3.31	7.74	3.47	.107	−0.09
Support by siblings	0–14	2.70	3.22	3.00	3.47	.053	0.11
Support by friends	0–14	4.12	3.38	4.72	3.46	.003	0.17
Support by teammates	0–14	4.53	3.94	5.20	3.83	<.001	0.20
Support by coach	0–14	5.38	4.07	5.62	3.64	.223	0.07
Support by PE teacher	0–14	3.10	3.15	2.75	2.90	.055	−0.11
Types of social support							
Emotional support	0–7	3.49	1.57	3.76	1.45	<.001	0.19
Esteem support	0–7	3.11	1.71	3.27	1.66	.066	0.11
Informational support	0–7	2.44	1.36	2.51	1.33	.324	0.06
Instrumental support	0–7	1.70	0.89	1.68	0.78	.700	−0.02
Co-participation	0–7	2.12	1.41	2.00	1.18	.108	−0.09
Modelling	0–7	1.34	1.48	1.14	1.32	.041	−0.12
Covariates							
Perceived competence	0–4	2.92	0.45	2.77	0.49	.047	−0.12
Motives	0–4	3.03	0.55	3.09	0.55	.023	0.13

### 3.2 Sources of social support

Table 4 presents the results of the multilevel linear fixed effects regression models for the sources of social support. In Model 1, the background variables age, gender, and ethnicity, and baseline participation significantly predicted adolescents' hours of participation in organized sports. Boys spent more time in organized sports than girls (*B* = 0.77, *p* < .01), and native Dutch adolescents spent more time in organized sports than non-native Dutch adolescents (*B* = 1.23, *p* < .01). Additionally, adolescents who participated in organized sports during their first year of secondary education participated more hours per week in organized sports (*B* = 2.30, *p* < .001). Finally, older adolescents spent less time in organized sports (*B* = −0.74, *p* < .05).

**Table 4:** Relationships between sources of social support and organized sports participation.

	Model 1	Model 2a	Model 2b	Model 2c	Model 2d	Model 2e	Model 2f	Model 2 g	Model 3	Model 4
Age	−0.74*	−0.76*	−0.66*	−0.69*	−0.70*	−0.62*	−0.59	−0.74*	−0.61*	−0.67*
	(0.32)	(0.32)	(0.32)	(0.32)	(0.31)	(0.31)	(0.31)	(0.32)	(0.31)	(0.31)
Boy	0.77**	0.73**	0.82**	0.82**	0.89**	0.75**	0.60*	0.77**	0.75**	0.56*
	(0.27)	(0.27)	(0.27)	(0.27)	(0.27)	(0.26)	(0.26)	(0.28)	(0.26)	(0.28)
Native	1.23**	0.98*	1.20**	1.27**	1.17**	0.95*	1.00*	1.22**	0.88*	0.98*
	(0.41)	(0.41)	(0.41)	(0.41)	(0.40)	(0.39)	(0.40)	(0.41)	(0.40)	(0.40)
BMI	−0.05	−0.05	−0.06	−0.05	−0.04	−0.04	−0.06	−0.05	−0.05	−0.05
	(0.05)	(0.05)	(0.05)	(0.05)	(0.05)	(0.05)	(0.05)	(0.05)	(0.05)	(0.05)
Sport (yes/no) in Year 1	2.30***	2.07***	2.23***	2.24***	2.27***	1.60***	1.31**	2.30***	1.11*	0.88*
	(0.42)	(0.42)	(0.42)	(0.42)	(0.41)	(0.41)	(0.43)	(0.42)	(0.43)	(0.44)
Year 3	1.05*	1.06*	1.00*	0.97*	0.93*	0.83*	0.91*	1.05*	1.11*	0.85*
	(0.44)	(0.44)	(0.44)	(0.44)	(0.43)	(0.42)	(0.42)	(0.44)	(0.43)	(0.41)
Support by father		0.10**							0.07	0.06
		(0.04)							(0.04)	(0.04)
Support by mother			0.07						−0.03	−0.04
			(0.04)						(0.05)	(0.04)
Support by siblings				0.06					0.00	−0.00
				(0.04)					(0.04)	(0.04)
Support by friends					0.16***				0.07	0.06
					(0.04)				(0.04)	(0.04)
Support by team						0.22***			0.12*	0.11*
						(0.03)			(0.05)	(0.05)
Support by coach							0.21***		0.17***	0.15**
							(0.04)		(0.05)	(0.05)
Support by teacher								0.01	−0.15**	−0.16***
								(0.04)	(0.05)	(0.05)
Perceived competence										0.39
										(0.30)
Motives										0.60*
										(0.27)
Intercept	11.43*	11.23*	9.98*	10.39*	10.07*	9.30*	9.54*	11.40*	9.31*	7.95
	(4.47)	(4.44)	(4.55)	(4.52)	(4.40)	(4.28)	(4.32)	(4.48)	(4.30)	(4.34)
R-squared for between model	9.1 × 10^−2^	8.9 × 10^−2^	.11	9.9 × 10^−2^	7.6 × 10^−2^	.18	.15	8.7 × 10^−2^	.24	.28
R-squared for within model	.13	.15	.14	.14	.17	.21	.2	.13	.25	.26

Table presents the regression coefficients and standard errors in brackets. The dummy variable 'school year' was used to model repeated measures; in the table, Year 3 represents the repeated measures.

Significance levels:

* *p* < .05.

** *p* < .01.

*** *p* < .001.

In Models 2a-g, we examined the relationships between the different sources of social support and adolescents' hours of participation in organized sports. When added separately, social support from fathers, friends, teammates, and coaches significantly predicted adolescents' hours of participation in organized sports. In contrast, social support from mothers, siblings, and PE teachers did not have a significant effect. Specifically, adolescents who reported higher levels of social support from their fathers (*B* = 0.10, *p* < .01), friends (*B* = 0.16, *p* < .001), teammates (*B* = 0.22, *p* < .001), and coaches (*B* = 0.21, *p* < .001) spent more time in organized sports. The higher explained variance between individuals for the models including social support from teammates (Between R-squared = 0.18) and coaches (Between R-squared = 0.15), compared to the models including support from fathers (Between R-squared = 0.09) and friends (Between R-squared = 0.08), suggests that social support from teammates as well as from coaches are stronger predictors of adolescents' participation in organized sports than social support from other sources.

In Model 3, where all sources of social support were included simultaneously, only social support from teammates (*B* = 0.12, *p* < .05) and coaches (*B* = 0.17, *p* < .001) remained significant predictors of adolescents' hours of participation in organized sports. Additionally, social support from PE teachers emerged as a significant predictor in this model. Specifically, adolescents who reported higher levels of social support from PE teachers spent less time in organized sports (B = −0.15, *p* < .01). The explained variance in this model, which included all sources of social support, was 0.24 (Between R-squared). This represents an increase compared to the explained variance in models that included only one source of social support at a time, suggesting that combining multiple sources of social support is more predictive of participation in organized sports.

In Model 4, after adding the covariates perceived competence and motives, social support from teammates (*B* = 0.11, *p* < .05), coaches (*B* = 0.15, *p* < .001), and PE teachers (*B* = −0.16, *p* < .01) continued to be significant predictors of adolescents' hours of participation in organized sports. Furthermore, motives (*B* = 0.60, *p* < .05) emerged as a significant predictor, while perceived competence (*B* = 0.39, *p* = .197) did not show statistical significance. The explained variance in this model (Between R-squared = 0.28) was slightly higher than in the previous model (Between R-squared = 0.24), suggesting only a marginal improvement in the model's predictive ability after adding the covariates.

In Models 5a-g and 6 (see Supplementary Table S1), we examined changes in the relationships between second and third year of secondary education by adding interaction terms between each source of social support and the school year of data collection. The models showed no significant changes over time in the relationships between the sources of social support and participation in organized sports. Furthermore, these models did not increase the explained variance compared to the previous models.

### 3.3 Types of social support

Table 5 presents the results of the multilevel linear fixed effects regression models for the types of social support. In Models 2a-g, we examined the relationships between the different types of social support and adolescents' hours of participation in organized sports. When added separately, all types of support except for modelling significantly predicted adolescents' hours of participation in organized sports. Specifically, adolescents who reported higher levels of emotional (*B* = 0.41, *p* < .001), esteem (*B* = 0.41, *p* < .001), informational (*B* = 0.38, *p* < .001), and instrumental support (B = 0.85, *p* < .001), and co-participation (*B* = 0.52, *p* < .001) spent more time in organized sports. The higher explained variance for the models including emotional (Between R-squared = 0.15) and instrumental support (Between R-squared = 0.15), compared to esteem (Between R-squared = 0.08), informational support (Between R-squared = 0.09), and co-participation (Between R-squared = 0.07), suggests that emotional and instrumental support are more robust predictors of adolescents' participation in organized sports compared to the other types of support.

**Table 5:** Relationships between types of social support and organized sports participation.

	Model 1	Model 2a	Model 2b	Model 2c	Model 2d	Model 2e	Model 2f	Model 3f	Model 4
Age	−0.74*	−0.64*	−0.69*	−0.62	−0.49	−0.59	−0.73*	−0.59	−0.67*
	(0.32)	(0.31)	(0.31)	(0.32)	(0.31)	(0.31)	(0.32)	(0.31)	(0.31)
Boy	0.77**	0.81**	0.66*	0.76**	0.86**	0.77**	0.78**	0.65*	0.49
	(0.27)	(0.27)	(0.27)	(0.27)	(0.26)	(0.27)	(0.28)	(0.26)	(0.27)
Native	1.23**	0.95*	0.97*	1.10**	1.00*	1.17**	1.23**	0.82*	0.94*
	(0.41)	(0.40)	(0.40)	(0.40)	(0.40)	(0.40)	(0.41)	(0.39)	(0.39)
BMI	−0.05	−0.04	−0.04	−0.05	−0.06	−0.07	−0.05	−0.05	−0.06
	(0.05)	(0.05)	(0.05)	(0.05)	(0.05)	(0.05)	(0.05)	(0.05)	(0.05)
Sport (yes/no) in Year 1	2.30***	1.82***	1.71***	1.96***	1.85***	1.80***	2.30***	1.30**	1.09*
	(0.42)	(0.42)	(0.42)	(0.42)	(0.41)	(0.41)	(0.42)	(0.41)	(0.42)
Year 3	1.05*	0.88*	0.95*	0.93*	0.80	0.93*	1.04*	0.83*	0.91*
	(0.44)	(0.43)	(0.43)	(0.43)	(0.43)	(0.43)	(0.44)	(0.42)	(0.41)
Emotional support		0.41***						0.10	0.08
		(0.09)						(0.13)	(0.13)
Esteem support			0.41***					0.28*	0.25
			(0.08)					(0.13)	(0.13)
Informational support				0.38***				−0.05	−0.08
				(0.10)				(0.14)	(0.14)
Instrumental support					0.85***			0.52*	0.43*
					(0.15)			(0.21)	(0.21)
Co-participation						0.52***		0.36**	0.34**
						(0.10)		(0.12)	(0.12)
Modelling							0.02	−0.37***	−0.37***
							(0.09)	(0.10)	(0.10)
Perceived competence									0.22
									(0.31)
Motives									0.65*
									(0.27)
Intercept	11.43*	9.02*	9.87*	9.13*	7.19	9.05*	11.28*	8.29	7.52
	(4.47)	(4.40)	(4.36)	(4.44)	(4.39)	(4.36)	(4.52)	(4.29)	(4.36)
R-squared for between model	9.1 × 10^−2^	.15	8.4 × 10^−2^	9.2 × 10^−2^	.15	7.3 × 10^−2^	8.9 × 10^−2^	.15	.19
R-squared for within model	.13	.17	.18	.16	.19	.19	.13	.24	.26

Table presents the regression coefficients and standard errors in brackets. The dummy variable 'school year' was used to model repeated measures; in the table, Year 3 represents the repeated measures.

Significance levels:

* *p* < .05.

** *p* < .01.

*** *p* < .001.

In Model 3, where all types of social support were included simultaneously, esteem (*B* = 0.28, *p* < .05), and instrumental support (*B* = 0.52, *p* < .05), and co-participation (*B* = 0.36, *p* < .001) remained significant predictors of adolescents' hours of participation in organized sports. In contrast, emotional (*B* = 0.10, *p* = 0.464) and informational support (*B* = −0.05, *p* = 0.716) were no longer significant predictors while modelling emerged as a significant predictor in this model (*B* = −0.37, *p* < .001). Specifically, adolescents who reported higher levels of modelling spent less time in organized sports. Interestingly, the explained variance between individuals in this model was equal to that of the models including emotional and instrumental support alone (Between R-squared = 0.15). The similar explained variance suggests that the inclusion of additional types of support beyond emotional or instrumental support did not improve the model's ability to predict adolescents' participation in organized sports.

In Model 4, after adding the covariates perceived competence and motives, esteem (*B* = 0.25, *p* < .05), and instrumental support (*B* = 0.43, *p* < .05), co-participation (*B* = 0.34, *p* < .01), and modelling (*B* = −0.37, *p* < .001) continued to be significant predictors of adolescents' hours of participation in organized sports. Additionally, motives (*B* = 0.65, *p* < .05) was a significant predictor, whereas perceived competence (*B* = 0.22, *p* = .468) was not. The explained variance in this model (Between R-squared = 0.19) was slightly higher than in the previous model (Between R-squared = 0.15).

In Models 5a-g and 6 (see Supplementary Table S2), we examined changes in the relationships between second and third year of secondary education by adding interaction terms between each type of social support and the school year of data collection. The models showed no significant changes over time in the relationships between the types of social support and participation in organized sports. Furthermore, these models showed no improvement in the explained variance compared to the previous model.

### 3.4 Individual variability

**Figure 1:**
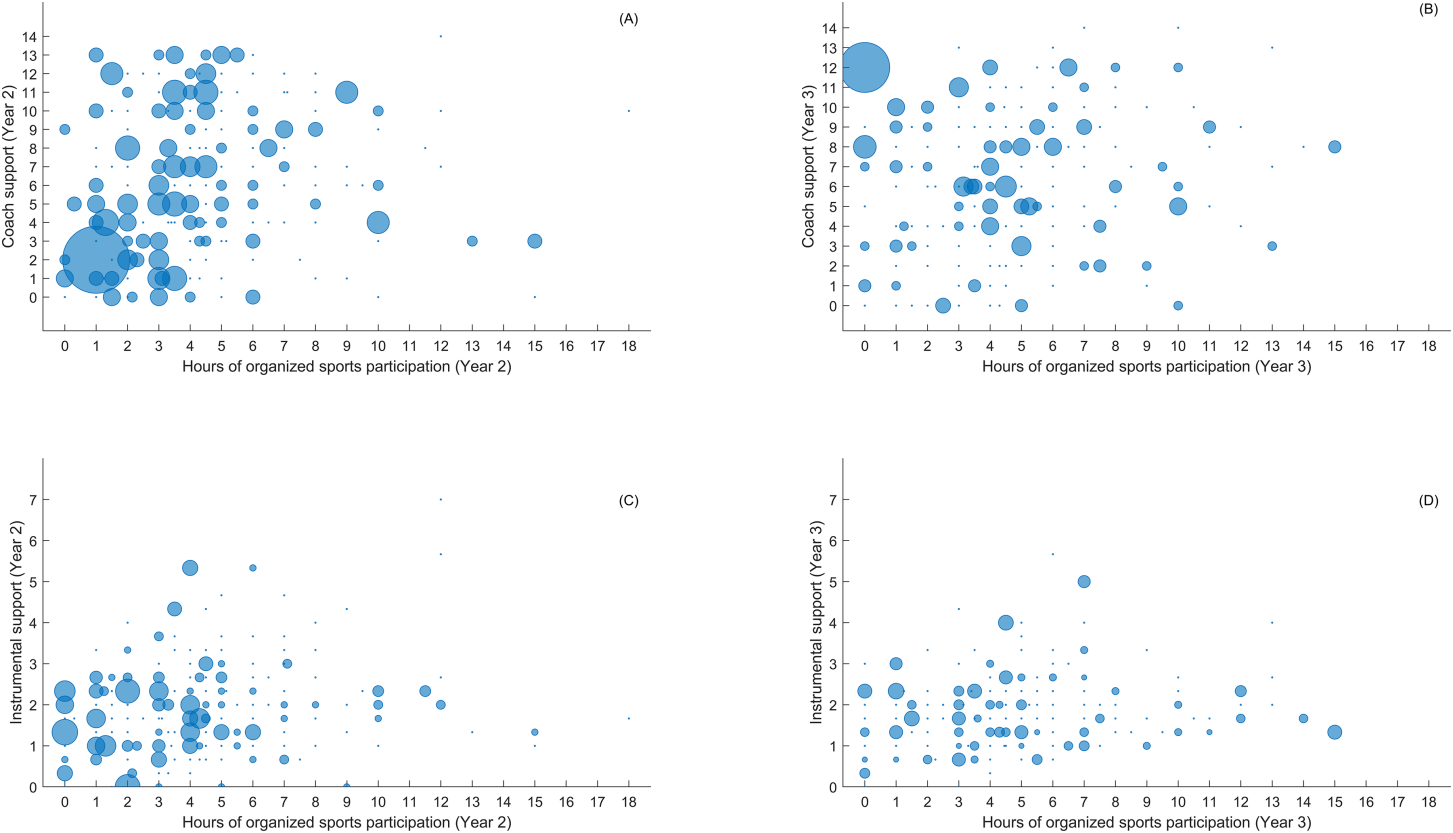
Individual variability in the relationship between sources and types of social support and hours of organized sports participation: **(A)** coach support at year 2, **(B)** coach support at year 3, **(C)** instrumental support at year 2, **(D)** instrumental support at year 3. The size of the circles in each panel reflects the number of participants, with the smallest circles representing 1 participant and the largest circles representing the maximum numbers of participants specific to each panel: 47, 35, 18, and 11 for panels A, B, C, and D, respectively.

Scatterplots were used to illustrate the relationships of the sources and types of social support with sports participation. Four representative scatterplots of these relationships are shown in Figures 1A–D, depicting coach support and instrumental support relative to hours spent in organized sports at both Year 2 and Year 3. On average, higher levels of coach and instrumental support were associated with more hours of sports participation (see Tables 4, 5). However, there was substantial variation between individuals. Some adolescents engage regularly in organized sports despite having low levels of social support, whereas others with low levels of support do no participate at all. Additionally, some adolescents participate rarely in organized sports despite high levels of social support, whereas others with high levels of social support participate regularly in organized sports. This variability indicates that the relationships between the sources and types of social support and sports participation are not uniform across individuals. Additional scatterplots illustrating these relationships can be found in the Supplementary Figures.

## 4 Discussion

Given the critical role of sports in promoting lifelong health and well-being, it is essential to understand the factors that influence adolescent sports participation, especially as it tends to decline during this period. To our knowledge, this study was the first to examine the association between different *sources* (i.e., father, mother, siblings, friends, teammates, coach, and PE teacher) and *types* (i.e., emotional, esteem, informational, instrumental, co-participation, modelling) of social support and adolescents' hours of participation in organized sports within a prospective study design. Our findings revealed significant relationships for various sources of social support and that social support from teammates and coaches consistently emerged as the strongest predictors of participation among adolescents in their second and third years of secondary education. Instrumental support also consistently predicted participation, while the relationships of the other types of support were smaller and less consistent. Additionally, we found that the relationships between the social support sources and types and sports participation remained stable over time between the second and third years of secondary education. Importantly, there was substantial individual variability, which indicates that the associations between the sources and types of social support and sports participation may not be uniform across all adolescents.

### 4.1 Sources of social support

Our results support previous research demonstrating the key role of teammates and coaches in adolescent sports participation (19, 21, 30, 48). Our findings also underscore the importance of additional sources of support (17). Specifically, although social support from parents, friends, siblings, and PE teachers appeared less significant, the combined impact of multiple sources was greater than the impact of each source individually. Notably, these relationships remained evident even after accounting for the covariates (i.e., motives and perceived competence). This highlights the need to consider multiple sources of social support to effectively promote organized sports participation.

Although earlier findings emphasized the positive influence of parental and peer support on adolescent sports participation (24), our results align with two studies indicating that teammates and coaches are more important than parents and friends during adolescence (19, 21). Specifically, the study of Chan et al. (21) indicated that while parents played a key role in childhood, the influence of teammates and coaches increased during adolescence. In a similar vein, the study by Gardner et al. (19) highlighted the central role of coaches and the less pronounced impact of parents and friends. Our study extends these insights by suggesting that the role of parents may be more general and less sport-specific, as they were reported as the primary sources of social support. Furthermore, we provide additional evidence that teammates, who are sometimes also friends, may play a more crucial role in sports participation compared to friends outside the sports environment. Overall, our findings suggest that sources within the sports environment—namely, teammates and coaches—play a more critical role in promoting sports participation among adolescents than those outside of this context.

Our study results further suggest a unique role for social support from PE teachers. Although PE teacher support alone was not significantly associated with organized sports participation, a negative relationship emerged when combined with other sources of support. Specifically, adolescents who spent more time in organized sports reported lower levels of PE teacher support but higher levels of support from teammates and coaches. In contrast, individuals who spent less time in organized sports reported higher levels of PE teacher support and lower levels of teammate and coach support. These combined findings suggest that support from PE teachers may serve as a compensatory resource when support from teammates or coaches is lacking, which aligns with the idea that some supportive relationships can compensate for the absence of others (19). Moreover, our results suggest that PE teacher support might be more influential in stimulating physical activities outside of organized sports. This is consistent with research showing that PE teachers can play a more pronounced role in other forms of physical activity, such as non-organized sports, cycling, and walking (20, 31). Thus, while PE teacher support may be less critical for adolescents regularly participating in organized sports, it might play a significant role in encouraging physical activity beyond this context, particularly for individuals who are less engaged in organized sports. Nevertheless, PE teachers should be mindful of how their support influences adolescents who are already regularly participating, as they have a responsibility to provide support to all students, including those who are highly engaged in sports.

### 4.2 Types of social support

The positive relationships that our study revealed between emotional, esteem, informational, instrumental support, and co-participation with sports participation align with existing literature (18, 23, 24). Additionally, our study results support the idea that multiple types of social support play significant roles in encouraging sports participation (17, 18, 24, 30). Nevertheless, instrumental support emerged as the most crucial, both as an individual predictor and within the combined model, with a substantial explained variance. This underscores its strong and consistent relationship with organized sports participation (23, 24) and is perhaps not very surprising given that transport and financial support are essential components of organized sports. Unlike other types of physical activity, organized sports often require transportation to and from practices and games, as well as financial investments for equipment, uniforms, and participation fees. These requirements make instrumental support critical for sustained involvement in organized sports, highlighting why it had such a strong relationship with adolescents' participation.

The effects of the other types of social support were more equivocal and varied based on the presence of other support types. Emotional support was a relative strong predictor when considered individually but less influential when considered alongside other types of support. This suggests that the relationship of emotional support with sports participation might be overshadowed by other types of support. This contrasts with earlier research that found stronger relationships for emotional support compared to other types of support, including instrumental support (26). However, this previous research focused on general physical activity rather than organized sports. Studies specifically on organized sports pointed to a more significant role for instrumental support (23, 24). Thus, while emotional support is likely crucial for overall physical activity, instrumental support may be more important for organized sports.

For esteem support, informational support, co-participation, and modelling, we showed only minimal impact in the present study. However, modelling emerged as a noteworthy factor in adolescent sports participation. It was identified as significant in the combined model, with a negative relationship with sports participation. Adolescents who reported higher levels of modelling tended to spend less time in organized sports, whereas those with lower levels of modelling spent more time in organized sports. Together, these findings suggest that the influence of each type of support is both context-dependent and interconnected (17, 19). This underscores the complex nature of social support in shaping adolescents' sports participation, which might also explain previous inconsistencies regarding the relationships of these types of support (17, 26, 27, 49). Further research is warranted to clarify these interactions and better understand the relative importance of different types of social support for adolescent sports participation.

### 4.3 Individual variability

While regression analyses revealed multiple significant relationships, the scatterplots provided further insight into the complex and nuanced nature of these relationships. The substantial individual variability depicted by the scatterplots suggests that adolescents' organized sports participation is not uniformly related to their levels of social support. While some individuals engage extensively without support, others do not participate despite high levels of support. This underscores the importance to consider individual differences when interpreting the results and suggests that a one-size-fits-all approach to stimulating sports participation through social support may be insufficient. Understanding the complex interplay of various types of support on an individual level is crucial for developing more personalized and effective strategies to encourage sports engagement. Future research using mixed-methods approaches are needed to more fully capture the individual experiences and contexts that influence how social support affects sports participation among adolescents. This approach would lead to an even more comprehensive understanding of these relationships, identifying for whom social support is effective and for whom it is not, thereby informing more tailored sport participation programs.

### 4.4 Changes in the relationships over one year

Interestingly, the relationships of the sources and types of social support with organized sports participation remained stable over the course of one year. This stability is notable given that adolescence is a period of significant individual (e.g., start of puberty) and environmental (e.g., new school, new peer groups) changes, which could potentially affect the relationship between social support and sports participation (50, 51). Evidence suggests that changes in social support can occur over periods of two years or more (32, 33), and these changes are associated with changes in physical activity levels (32). Our study builds on these findings by demonstrating that the strength of the relationship between social support and organized sports participation does not necessarily change over a shorter timeframe of one year. This suggests that while the amount of social support may vary, its impact on sports participation can remain stable. However, it is important to note that earlier studies examined longer durations, so our one-year study might have been insufficient to capture significant changes or may reflect a particularly stable period in adolescent development (33). To better understand how social support and organized sports participation interact, future research should consider tracking these factors over longer periods during adolescence.

### 4.5 Limitations

One limitation of the present study concerns the representativity of the sample. The high participation rate in organized sports among our participants, which ranged from 87%–88%, is considerably higher than the national average of 63% (8). This discrepancy may be partly due to the participating schools' emphasis on sports, which likely attracts students with higher engagement or motivation. Additionally, the sample included a low percentage of adolescents in pre-vocational secondary education (52), a group with generally lower sports participation rates (53). This limited representation may partly explain the higher rates of organized sports participation observed in our study. Future research should aim for a more representative sample, including more adolescents who do not participate in sports and/or are enrolled in pre-vocational secondary education.

Another limitation of our study involves the assessment of social support. We adapted the items related to emotional, esteem, informational, and instrumental support from previously validated questionnaires (28, 29). While these questionnaires effectively measure different types of social support, they do not distinguish between the sources of support. To address this, we included sources of social support but simplified the assessment by using binary items instead of frequency measures. This simplification aimed to reduce participant burden and minimize the time required for completing the assessment. However, this approach may have missed nuances in changes in social support and its relationship with sports participation, potentially impacting the depth of our results. Additionally, the use of single items to measure co-participation and modelling may have introduced limitations, as single-item measures often lack the depth and reliability of multi-item scales (see for example (54).

A third limitation of this study relates to the design and analysis of our data. We employed a longitudinal design to examine the relationships between the sources and types of social support and organized sports participation on two occasions over time. However, the relationships at both occasions were analyzed cross-sectionally, which precludes establishing causal relationships between the sources and types of social support and sports participation. Despite this limitation, the associations found in this study generally aligned with expected directions and underscored the relevance of different sources and types of social support, as well as the complex interactions between them. Further research using more robust longitudinal methodologies is warranted.

### 4.6 Future research

Our results revealed the complex role of social support in organized sports participation among adolescents. To better understand this complexity, future research should consider examining individual patterns more closely. This can be achieved by integrating both qualitative and quantitative approaches. Qualitative approaches can help identify the most significant combinations of sources and types of social support (17, 18, 26), which is crucial for the design of questionnaires that are both comprehensive and manageable. They are also crucial to identify which individuals would benefit from social support for sports participation and which do not. They enable researchers to explore the nuanced effects of non-modifiable factors like ethnicity and gender, as well as modifiable factors like motives and perceived competence. Complementing qualitative approaches, quantitative methods should prospectively track changes in social support and sports participation throughout adolescence. This comprehensive approach could guide interventions by targeting the most effective sources and types of social support tailored to individual patterns, thereby promoting sports participation among adolescents.

### 4.7 Practical implications

Based on our findings, we offer several practical recommendations for supporting adolescents in organized sports participation. These suggestions focus on the critical role of professionals in sports and PE settings. First, professionals in these environments should be mindful of individual differences among adolescents and tailor their approaches accordingly. To do so effectively, we advise that they engage directly and regularly with students during sports training, competitions, and PE lessons (55). Second, we highlight the essential responsibility of coaches and trainers, and suggest that they should be equipped with the pedagogical skills needed to foster a positive team culture and build strong relationships (56, 57). These practices can contribute to both effective team and coach support. Third, given the critical importance of instrumental support, it is crucial to assist adolescents whose parents are unable or unwilling to provide this type of support. PE teachers can play a pivotal role for these adolescents as they interact with all students regularly through school and PE classes, positioning them uniquely to identify those lacking instrumental support. An important first step, therefore, is for teachers to gain a clear understanding of their students' involvement in sports. Once they identify those facing barriers due to insufficient instrumental support, teachers can connect them with available resources, such as financial assistance for sports fees. Additionally, PE teachers can collaborate with local municipalities and sports clubs to develop inclusive programs that address the financial and logistical barriers families often face. By adopting these practices, sports and PE professionals may help create supportive environments that foster long-term sports participation.

## 5 Conclusions

This study enhances our understanding of the role of social support in organized sports participation among adolescents. Our results highlight the importance of social support from multiple sources, with teammates and coaches emerging as particularly influential. Furthermore, instrumental support was identified as a crucial type of social support, while the role of other types of social support appeared more nuanced. Notably, the significance of specific sources and types of social support varies among individuals, which indicates that a one-size-fits-all approach may not be effective. It is therefore essential to promote social support from within the sports environment and to encourage instrumental support, while also recognizing the need for tailored approaches that address individual differences. Future research should explore the individual differences and the complex interactions among different sources and types of social support using mixed methods and prospective designs. Such research could inform interventions strategies that stimulate continuous participation and prevent dropout in youth sports.

## Data Availability

The original datasets presented in the study are publicly available. This data can be found here: https://doi.org/10.17026/SS/LA3KIZ.
