# Supplementary material for: The key roles of teammates, coaches, and instrumental support in adolescent sports participation: a one-year prospective study
Source: Front Sports Act Living. 2025 Jan 20;7:1499693. doi: 10.3389/fspor.2025.1499693 (PMC11788341; doi:10.3389/fspor.2025.1499693)
Supplement: Supplementary file 1 [file Datasheet1.pdf]

Supplementary Figures

Each figure shows individual variability in the relationship between a specific source or type of social support hours and organized sports participation at either Year 2 or Year 3. The size of the circles in each plot reflects the number of participants, with the smallest circles representing 1 participant and the largest circles representing the maximum number of participants specific to each figure.

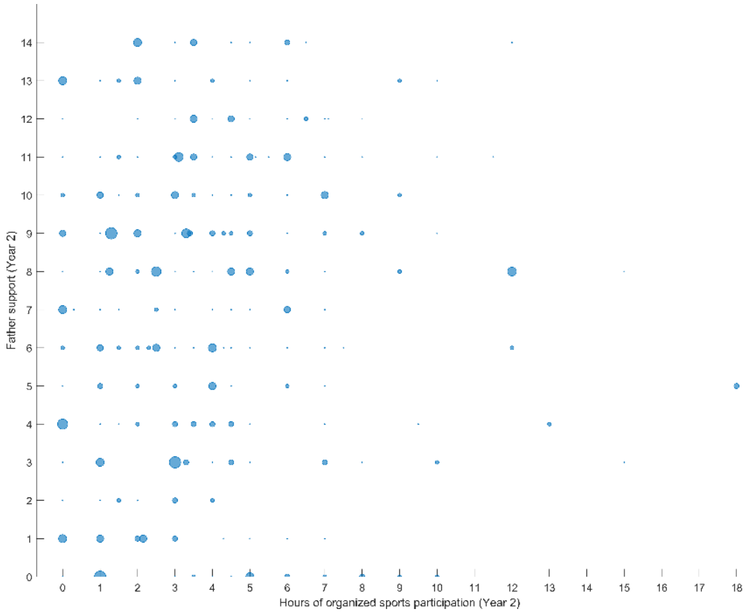

Figure S1. Father support at Year 2. Circle size ranges from 1 to 11 participants.

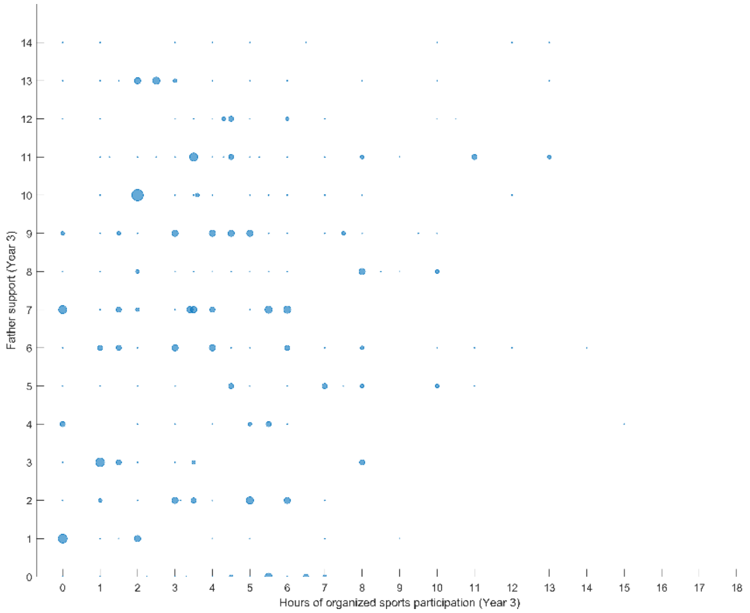

Figure S2. Father support at Year 3. Circle size ranges from 1 to 11 participants.

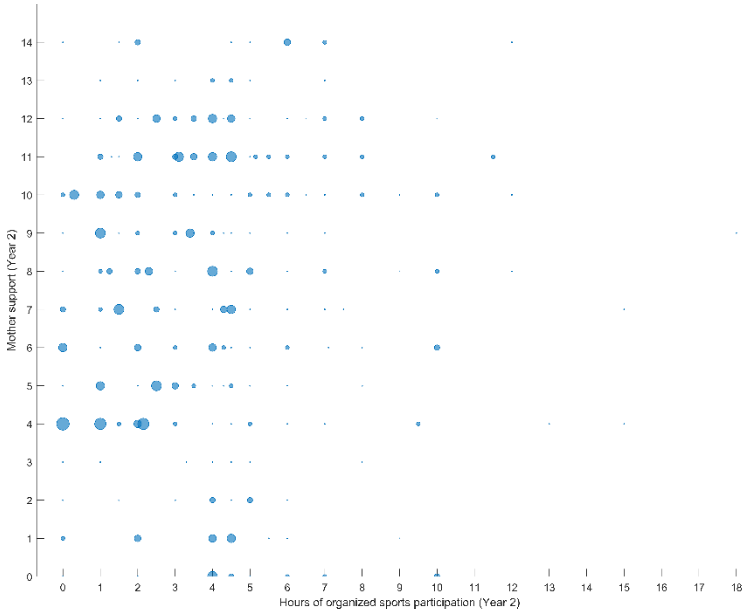

Figure S3. Mother support at Year 2. Circle size ranges from 1 to 12 participants.

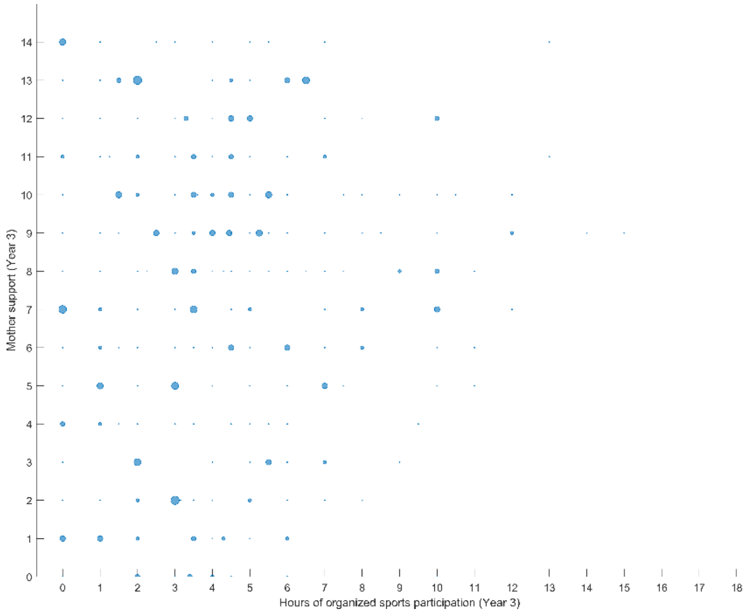

Figure S4. Mother support at Year 3. Circle size ranges from 1 to 8 participants.

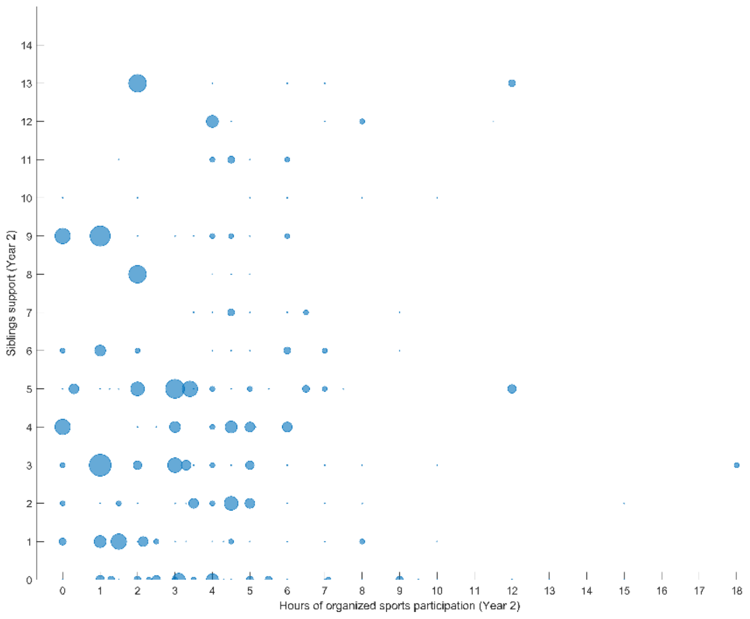

Figure S5. Siblings support at Year 2. Circle size ranges from 1 to 21 participants.

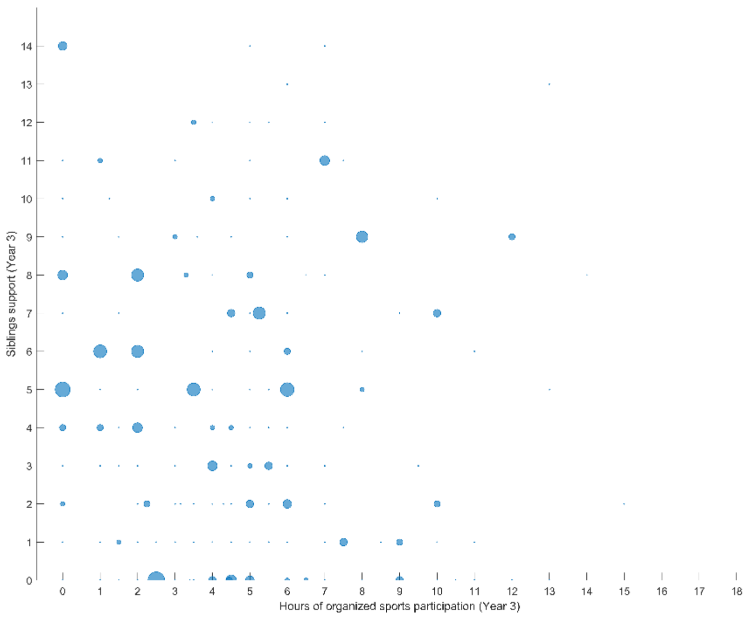

Figure S6. Siblings support at Year 3. Circle size ranges from 1 to 16 participants.

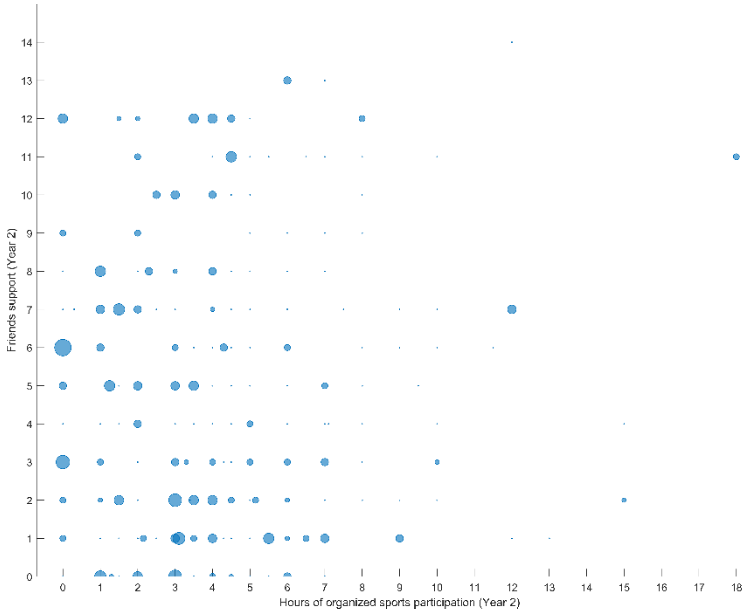

Figure S7. Friends support at Year 2. Circle size ranges from 1 to 16 participants.

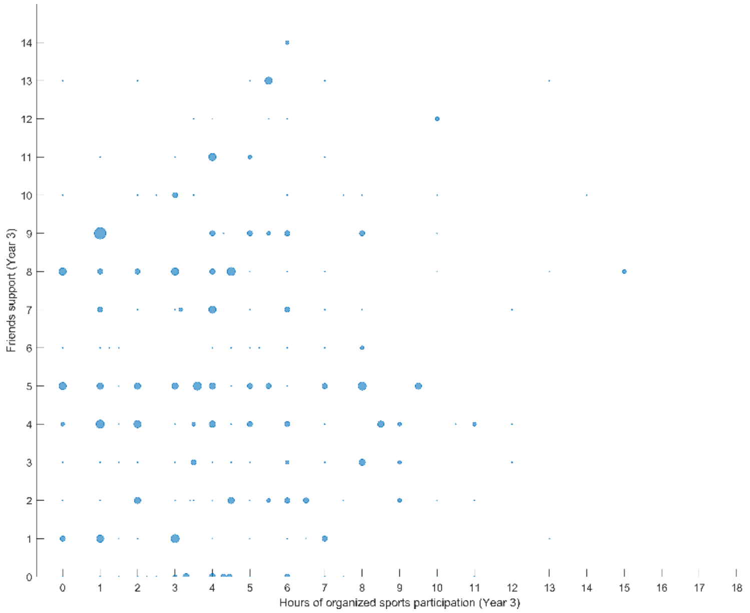

Figure S8. Friends support at Year 3. Circle size ranges from 1 to 11 participants.

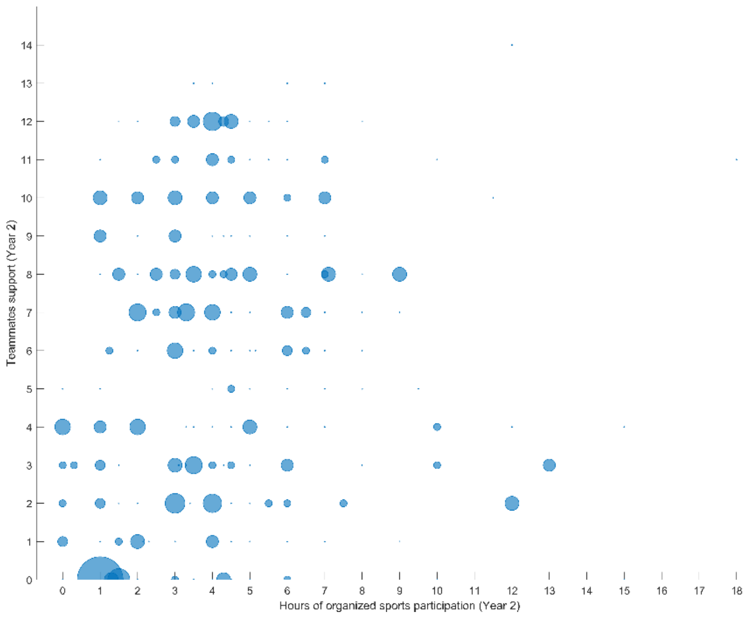

Figure S9. Teammates support at Year 2. Circle size ranges from 1 to 44 participants.

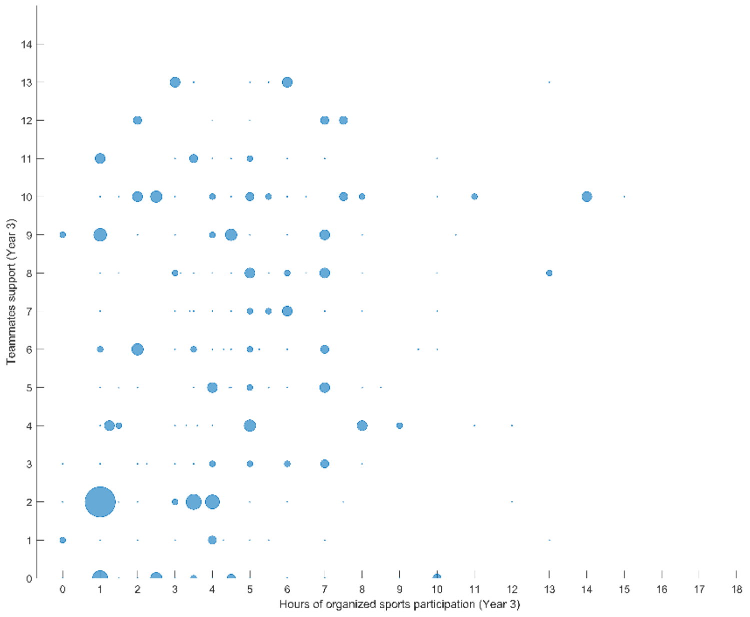

Figure S10. Teammates support at Year 3. Circle size ranges from 1 to 29 participants.

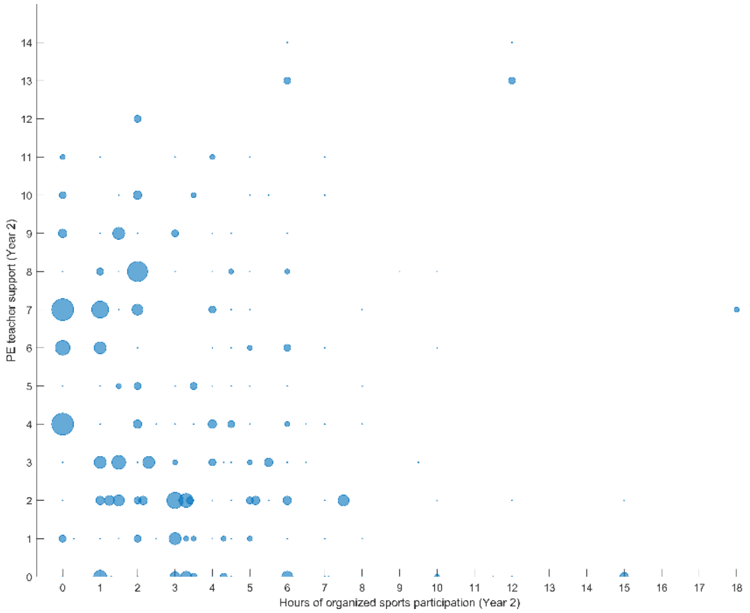

Figure S11. PE teacher support at Year 2. Circle size ranges from 1 to 21 participants.

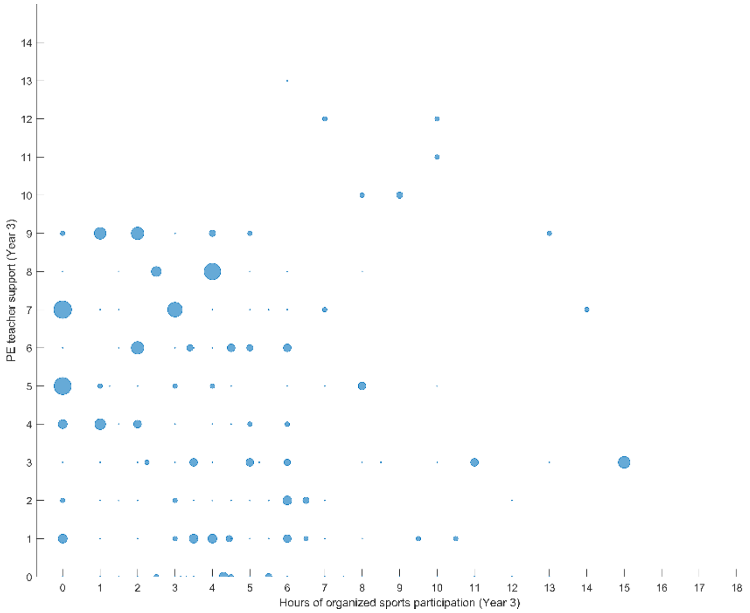

Figure S12. PE teacher support at Year 3. Circle size ranges from 1 to 17 participants.

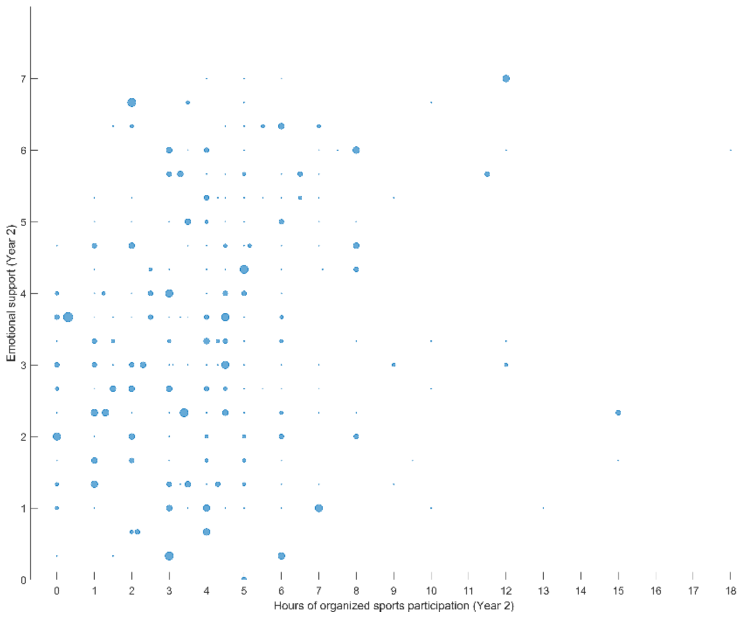

Figure S13. Emotional support at Year 2. Circle size ranges from 1 to 9 participants.

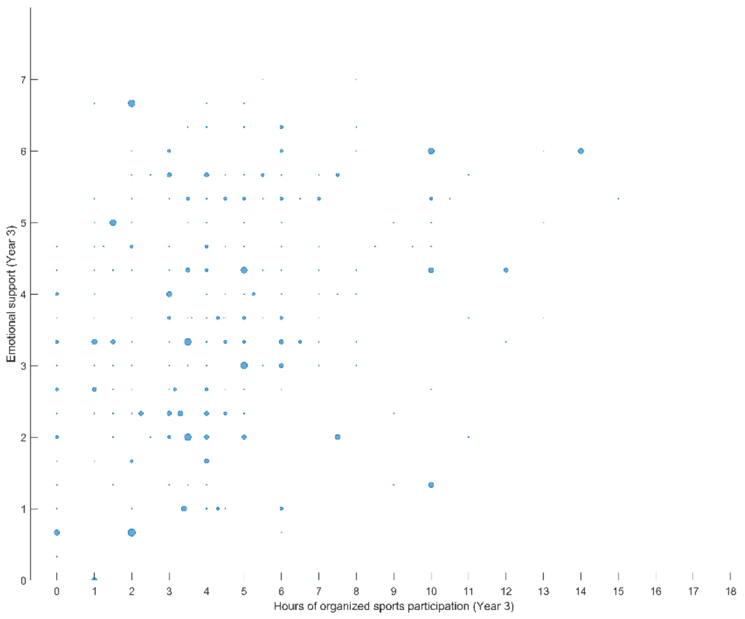

Figure S14. Emotional support at Year 3. Circle size ranges from 1 to 7 participants.

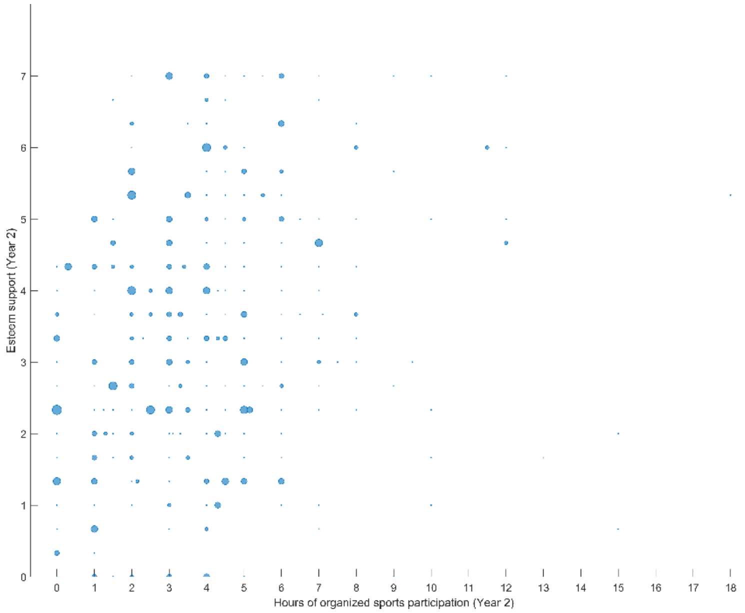

Figure S15. Esteem support at Year 2. Circle size ranges from 1 to 9 participants.

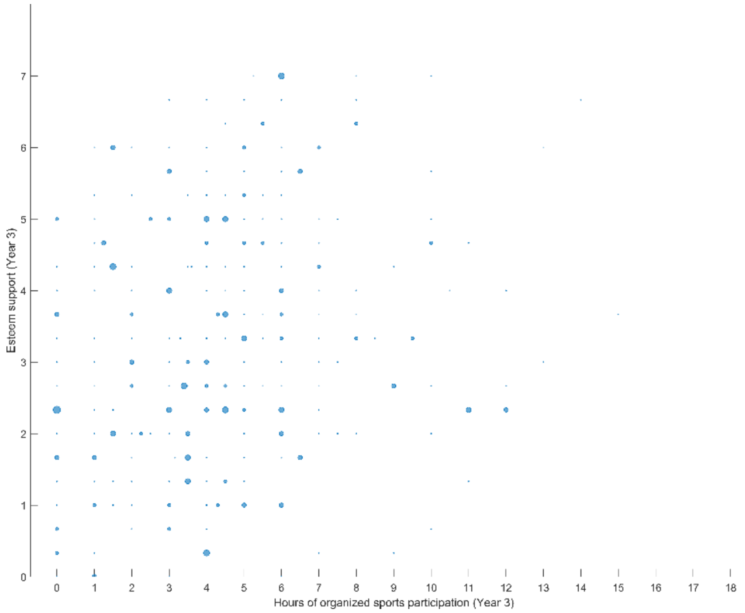

Figure S16. Esteem support at Year 3. Circle size ranges from 1 to 7 participants.

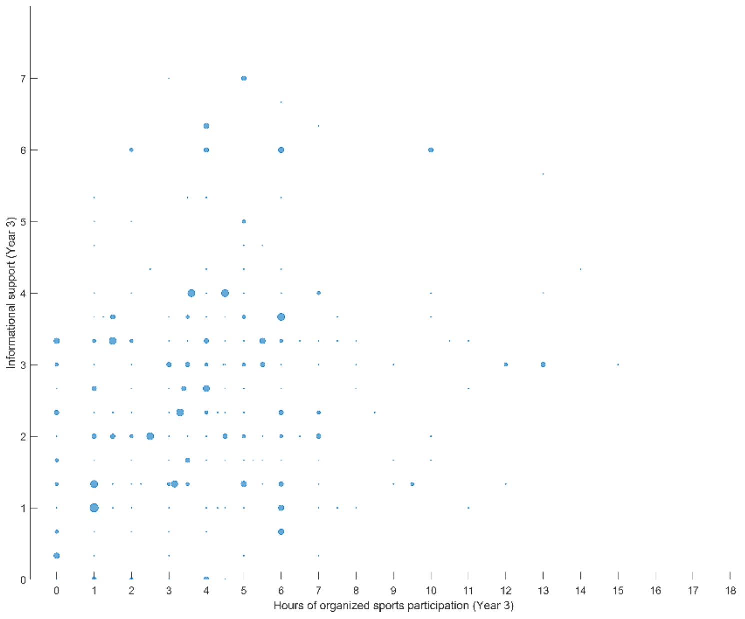

Figure S17. Informational support at Year 2. Circle size ranges from 1 to 11 participants.

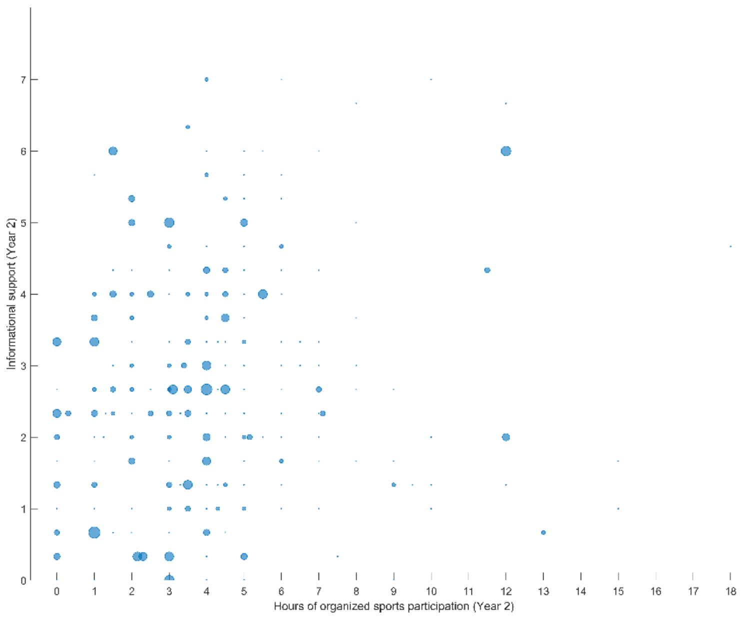

Figure S18. Informational support at Year 3. Circle size ranges from 1 to 8 participants.

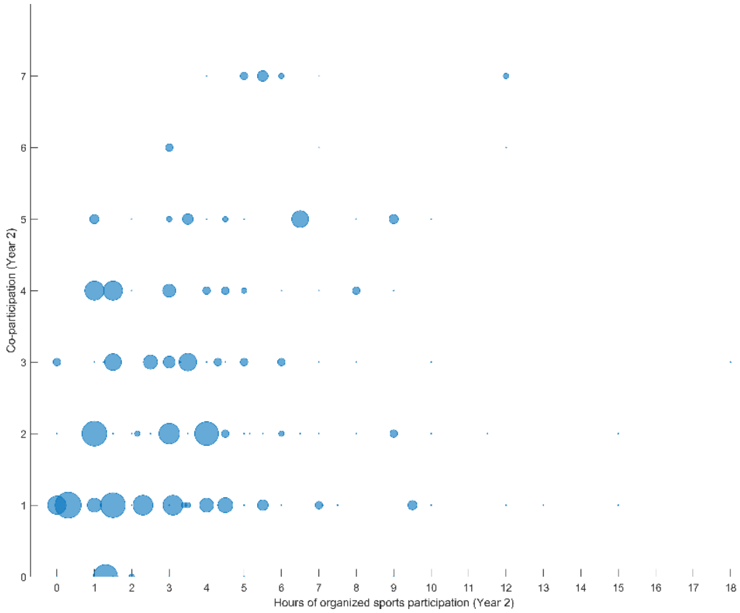

Figure S19. Co-participation at Year 2. Circle size ranges from 1 to 25 participants.

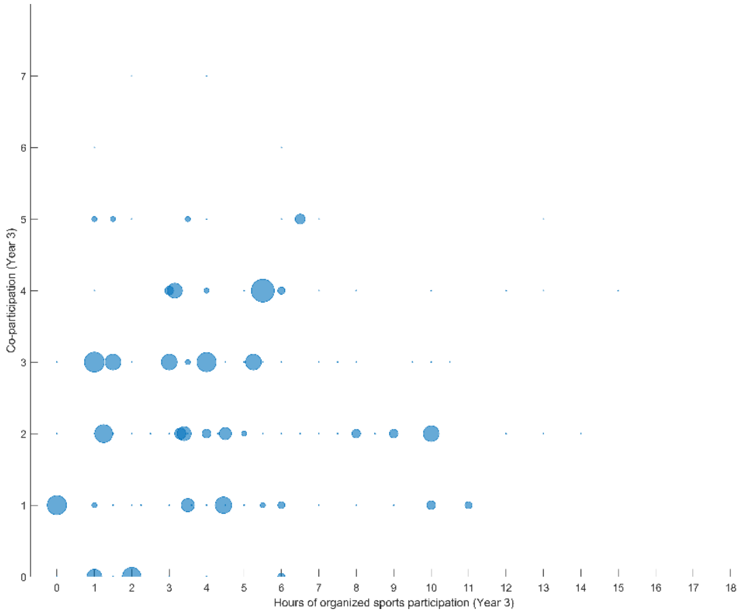

Figure S20. Co-participation at Year 3. Circle size ranges from 1 to 22 participants.

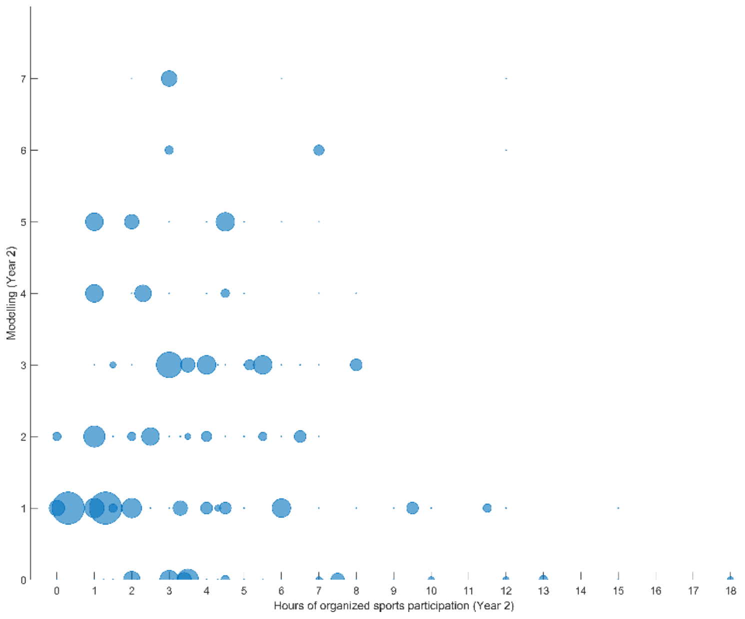

Figure S21. Modelling at Year 2. Circle size ranges from 1 to 31 participants.

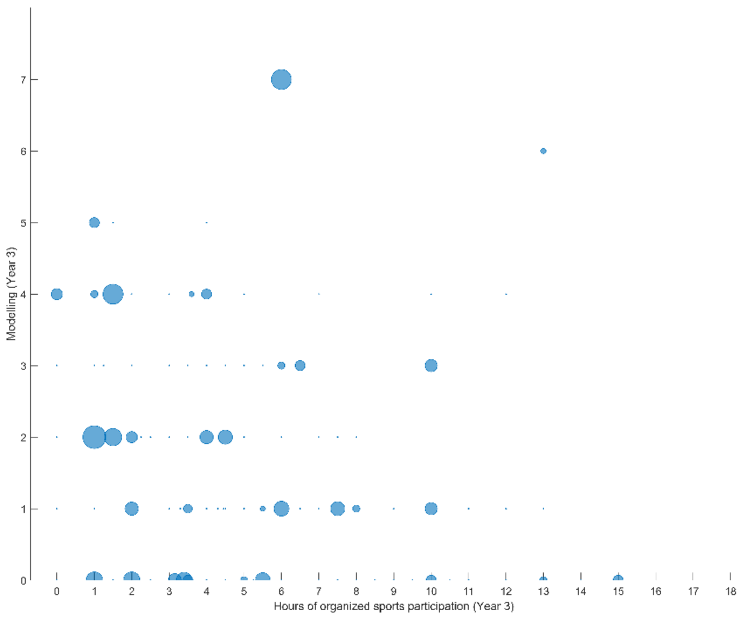

Figure S22. Modelling at Year 3. Circle size ranges from 1 to 22 participants.
