# Supplementary material for: The key roles of teammates, coaches, and instrumental support in adolescent sports participation: a one-year prospective study
Source: Front Sports Act Living. 2025 Jan 20;7:1499693. doi: 10.3389/fspor.2025.1499693 (PMC11788341; doi:10.3389/fspor.2025.1499693)
Supplement: Supplementary file 2 [file Datasheet2.pdf]

Supplementary Tables

Tables S1 and S2 present the relationships between social support and organized sports participation over time. The main effects of the sources/types of social support indicate the regression coefficient at Year 2, providing a baseline for the relationship at this time point. The interaction terms reflect the difference in the regression coefficients between Year 2 and Year 3, showing how the relationship changes over time.

Table S1. Changes in the relationships between sources of social support and organized sports participation over time

	Model 5a	Model 5b	Model 5c	Model 5d	Model 5e	Model 5f	Model 5g	Model 6
Age	-0.68 *	-0.68 *	-0.66 *	-0.67 *	-0.69 *	-0.67 *	-0.71 *	-0.73 *
	(0.31)	(0.31)	(0.31)	(0.31)	(0.31)	(0.31)	(0.31)	(0.31)
Boy	0.57 *	0.57 *	0.57 *	0.57 *	0.56 *	0.56 *	0.57 *	0.60 *
	(0.28)	(0.28)	(0.28)	(0.28)	(0.28)	(0.28)	(0.28)	(0.28)
Native	0.98 *	0.97 *	0.98 *	0.97 *	0.97 *	0.97 *	0.95 *	0.95 *
	(0.40)	(0.40)	(0.40)	(0.40)	(0.40)	(0.40)	(0.40)	(0.40)
BMI	-0.05	-0.05	-0.06	-0.05	-0.06	-0.06	-0.06	-0.06
	(0.05)	(0.05)	(0.05)	(0.05)	(0.05)	(0.05)	(0.05)	(0.05)
Sport (yes/no) in Year 1	0.88 *	0.88 *	0.88 *	0.89 *	0.86	0.86	0.88 *	0.89 *
	(0.44)	(0.44)	(0.44)	(0.44)	(0.44)	(0.44)	(0.44)	(0.44)
Year 3	0.64	0.55	0.95 *	0.98	0.64	0.69	0.58	0.58
	(0.64)	(0.71)	(0.46)	(0.51)	(0.50)	(0.53)	(0.47)	(0.79)
Support by father	0.04	0.06	0.06	0.06	0.06	0.06	0.06	0.07
	(0.05)	(0.04)	(0.04)	(0.04)	(0.04)	(0.04)	(0.04)	(0.06)
Support by mother	-0.04	-0.06	-0.04	-0.04	-0.04	-0.04	-0.05	-0.07
	(0.04)	(0.06)	(0.05)	(0.05)	(0.04)	(0.05)	(0.05)	(0.07)
Support by siblings	-0.00	-0.00	0.02	0.00	0.00	-0.00	0.00	0.03
	(0.04)	(0.04)	(0.06)	(0.04)	(0.04)	(0.04)	(0.04)	(0.06)
Support by friends	0.06	0.06	0.06	0.08	0.06	0.06	0.06	0.11
	(0.04)	(0.04)	(0.04)	(0.06)	(0.04)	(0.04)	(0.04)	(0.06)
Support by team	0.11 *	0.11 *	0.10 *	0.11 *	0.08	0.11 *	0.11 *	0.06
	(0.05)	(0.05)	(0.05)	(0.05)	(0.06)	(0.05)	(0.05)	(0.07)
Support by coach	0.15 **	0.15 **	0.15 **	0.15 **	0.15 **	0.14 *	0.15 **	0.18 **
	(0.05)	(0.05)	(0.05)	(0.05)	(0.05)	(0.05)	(0.05)	(0.07)
Support by teacher	-0.16 ***	-0.16 ***	-0.16 ***	-0.16 ***	-0.16 ***	-0.16 ***	-0.21 ***	-0.22 ***

	(0.05)	(0.05)	(0.05)	(0.05)	(0.05)	(0.05)	(0.06)	(0.07)
Perceived competence	0.39	0.39	0.38	0.38	0.38	0.38	0.38	0.35
	(0.30)	(0.30)	(0.30)	(0.30)	(0.30)	(0.30)	(0.30)	(0.30)
Motives	0.59 *	0.60 *	0.60 *	0.60 *	0.59 *	0.60 *	0.59 *	0.60 *
	(0.27)	(0.27)	(0.27)	(0.27)	(0.27)	(0.27)	(0.27)	(0.27)
Year 3 # Support by father	0.03							-0.01
	(0.06)							(0.07)
Year 3 # Support by mother		0.04						0.05
		(0.07)						(0.09)
Year 3 # Support by siblings			-0.03					-0.04
			(0.07)					(0.08)
Year 3 # Support by friends				-0.03				-0.08
				(0.07)				(0.09)
Year 3 # Support by team					0.05			0.09
					(0.06)			(0.09)
Year 3 # Support by coach						0.03		-0.05
						(0.06)		(0.09)
Year 3 # Support by teacher							0.10	0.11
							(0.08)	(0.09)
Intercept	8.12	8.17	7.79	7.87	8.33	8.12	8.66 *	8.95 *
	(4.36)	(4.37)	(4.36)	(4.35)	(4.37)	(4.36)	(4.37)	(4.43)
R-squared for between model	.28	.28	.29	.29	.27	.28	.28	.28
R-squared for within model	.26	.26	.26	.26	.27	.26	.27	.27

Table presents the regression coefficients and standard errors in brackets. The dummy variable 'school year' was used to model repeated measures; in the table, Year 3 represents the repeated measures. Significance levels: *p<.05; **p<.01; ***p<.001.

Table S2. Changes in the relationships between types of social support and organized sports participation over time

	Model 5a	Model 5b	Model 5c	Model 5d	Model 5e	Model 5f	Model 6
Age	-0.68 *	-0.67 *	-0.68 *	-0.67 *	-0.67 *	-0.67 *	-0.66 *
	(0.31)	(0.31)	(0.31)	(0.31)	(0.31)	(0.31)	(0.31)
Boy	0.49	0.49	0.50	0.49	0.49	0.49	0.50
	(0.27)	(0.27)	(0.27)	(0.27)	(0.27)	(0.27)	(0.27)
Native	0.94 *	0.94 *	0.93 *	0.93 *	0.94 *	0.94 *	0.93 *
	(0.39)	(0.39)	(0.39)	(0.39)	(0.39)	(0.39)	(0.40)
BMI	-0.06	-0.06	-0.06	-0.06	-0.06	-0.06	-0.06
	(0.05)	(0.05)	(0.05)	(0.05)	(0.05)	(0.05)	(0.05)
Sport (yes/no) in Year 1	1.08 *	1.09 *	1.08 *	1.09 *	1.08 *	1.09 *	1.06 *
	(0.42)	(0.42)	(0.42)	(0.42)	(0.42)	(0.42)	(0.42)
Year 3	0.71	0.93	0.65	0.96	0.70	0.95 *	0.68
	(0.72)	(0.60)	(0.59)	(0.63)	(0.56)	(0.47)	(0.76)
Emotional support	0.06	0.08	0.09	0.08	0.09	0.08	0.03
	(0.15)	(0.14)	(0.13)	(0.14)	(0.14)	(0.14)	(0.18)
Esteem support	0.25	0.25	0.25	0.25	0.25	0.25	0.34
	(0.13)	(0.15)	(0.13)	(0.13)	(0.13)	(0.13)	(0.18)
Informational support	-0.08	-0.08	-0.13	-0.08	-0.08	-0.08	-0.21
	(0.14)	(0.14)	(0.17)	(0.14)	(0.14)	(0.14)	(0.20)
Instrumental support	0.43 *	0.43 *	0.44 *	0.45	0.44 *	0.43 *	0.55
	(0.21)	(0.21)	(0.21)	(0.24)	(0.21)	(0.21)	(0.28)
Co-participation	0.34 **	0.34 **	0.35 **	0.34 **	0.30 *	0.34 **	0.29
	(0.12)	(0.12)	(0.12)	(0.12)	(0.14)	(0.12)	(0.15)
Modelling	-0.37 ***	-0.37 ***	-0.36 ***	-0.37 ***	-0.37 ***	-0.35 **	-0.32 *
	(0.10)	(0.10)	(0.10)	(0.10)	(0.10)	(0.13)	(0.14)
Perceived competence	0.22	0.23	0.23	0.22	0.23	0.22	0.23
	(0.31)	(0.31)	(0.31)	(0.31)	(0.31)	(0.31)	(0.31)
Motives	0.65 *	0.65 *	0.64 *	0.65 *	0.64 *	0.65 *	0.66 *
	(0.27)	(0.27)	(0.27)	(0.27)	(0.27)	(0.27)	(0.27)
Year 3 # Emotional support	0.05						0.11
	(0.16)						(0.27)
Year 3 # Esteem support		-0.01					-0.17
		(0.14)					(0.24)
Year 3 # Informational support			0.11				0.26
			(0.18)				(0.29)
Year 3 # Instrumental support				-0.03			-0.26
				(0.29)			(0.41)

Year 3 # Co-participation					0.10<br>(0.19)		0.14<br>(0.23)
Year 3 # Modelling						-0.03<br>(0.17)	-0.09<br>(0.20)
Intercept	7.65<br>(4.38)	7.49<br>(4.41)	7.80<br>(4.39)	7.48<br>(4.38)	7.58<br>(4.37)	7.52<br>(4.37)	7.47<br>(4.44)
R-squared for between model	.19	.19	.18	.19	.18	.18	.16
R-squared for within model	.26	.26	.26	.26	.26	.26	.26

Table presents the regression coefficients and standard errors in brackets. The dummy variable 'school year' was used to model repeated measures; in the table, Year 3 represents the repeated measures. Significance levels: *p<.05; **p<.01; ***p<.001.
